# WHEN THE LIGHTS WENT OUT AT ADULT DAY CENTERS: CAREGIVER WELL-BEING DURING THE COVID-19 LOCKDOWNS

**DOI:** 10.1093/geroni/igac059.2969

**Published:** 2022-12-20

**Authors:** Rachael Turner, Celinda Reese-Melancon, Erin Harrington, Micaela Andreo

**Affiliations:** Oklahoma State University, Stillwater, Oklahoma, United States; Oklahoma State University, Stillwater, Oklahoma, United States; Pennsylvania State University, University Park, Pennsylvania, United States; University of Texas at Dallas, Dallas, Texas, United States

## Abstract

Adult Day Centers (ADC) are community-based programs that provide services to adults with dementia or other disabilities. Unfortunately, many ADCs were closed during the early stages of the COVID-19 pandemic, leaving caregivers without an important resource. The current study sought to identify novel challenges that caregivers experienced with the closure of ADCs. From May to July 2020, caregivers were recruited from ADCs across the United States and completed an online survey regarding ADC use, feelings of caregiver burden, perceived stress, and perceived change in these variables and others (i.e., time spent caregiving, caregiver and care recipient memory) resulting from the pandemic. Qualitative reports of additional pandemic-related caregiving experiences were also captured. The current work included 70 caregivers, most of whom were caring for a family member with dementia. Correlational analyses indicated that more frequent ADC use prior to the pandemic was associated with reporting an increase in the amount of time spent on caregiving tasks, an increase in caregiver burden, and increased feelings of stress since the onset of the pandemic. Prior use was not related to change in perceptions of caregiver forgetfulness or changes in care recipient memory behaviors. Prominent themes from caregivers’ qualitative reports included mental health concerns, apprehension regarding the virus itself, and the need for respite. These findings support theories of caregiver stress (Pearlin et al., 1990) and provide insight into how caregivers were impacted by the loss of a key resource to support their caregiving.

